# Rapid Response of Hydrological Loss of DOC to Water Table Drawdown and Warming in Zoige Peatland: Results from a Mesocosm Experiment

**DOI:** 10.1371/journal.pone.0109861

**Published:** 2014-11-04

**Authors:** Xue-Dong Lou, Sheng-Qiang Zhai, Bing Kang, Ya-Lin Hu, Li-Le Hu

**Affiliations:** 1 Chinese Research Academy of Environmental Sciences, Beijing, China; 2 College of Life Sciences, Northwest Agriculture & Forestry University, Yangling, Shaanxi, China; 3 Institute of Applied Ecology, Chinese Academy of Sciences, Shenyang, China; Institute of Tibetan Plateau Research, China

## Abstract

A large portion of the global carbon pool is stored in peatlands, which are sensitive to a changing environment conditions. The hydrological loss of dissolved organic carbon (DOC) is believed to play a key role in determining the carbon balance in peatlands. Zoige peatland, the largest peat store in China, is experiencing climatic warming and drying as well as experiencing severe artificial drainage. Using a fully crossed factorial design, we experimentally manipulated temperature and controlled the water tables in large mesocosms containing intact peat monoliths. Specifically, we determined the impact of warming and water table position on the hydrological loss of DOC, the exported amounts, concentrations and qualities of DOC, and the discharge volume in Zoige peatland. Our results revealed that of the water table position had a greater impact on DOC export than the warming treatment, which showed no interactive effects with the water table treatment. Both DOC concentration and discharge volume were significantly increased when water table drawdown, while only the DOC concentration was significantly promoted by warming treatment. Annual DOC export was increased by 69% and 102% when the water table, controlled at 0 cm, was experimentally lowered by −10 cm and −20 cm. Increases in colored and aromatic constituents of DOC (measured by Abs_254 nm_, SUVA_254 nm_, Abs_400 nm_, and SUVA_400 nm_) were observed under the lower water tables and at the higher peat temperature. Our results provide an indication of the potential impacts of climatic change and anthropogenic drainage on the carbon cycle and/or water storage in a peatland and simultaneously imply the likelihood of potential damage to downstream ecosystems. Furthermore, our results highlight the need for local protection and sustainable development, as well as suggest that more research is required to better understand the impacts of climatic change and artificial disturbances on peatland degradation.

## Introduction

Generally, peat-accumulating wetlands provide waterlogged conditions where carbon accumulation is encouraged [1], and therefore have huge carbon storage potential. However, there is increasing concern that carbon storage in peatlands is unstable and may be susceptible to water table drawdown and higher temperatures over the next two centuries due to projected climatic change [2]–[6]. Furthermore, the water table in peatlands may also be significantly lowered by drainage resulting from human activities [7], [8]. As the largest highland wetland in the world [9] and the largest peat storage area in China, the Zoige alpine wetland serves as a natural barrier and prevents desertification in Northwest China, extending farther toward Southeast China, and is very sensitive to climate change [10]. The peatland in Zoige is also the major water source of the world's largest plateau reserve (i.e., Three-Rivers Source Nature Reserve), supplying water for the three most important rivers in East Asia (i.e. the Yellow, Yangtze, and Lancang rivers) [11]. The Zoige wetland is particularly closely associated with the ecological security of the Yellow River drainage basin [12] because it provides about 40% of the total flow of the Yellow River [13]. Zoige peatland covers an estimated area of 0.5 million hectares and accounts for 47.53% of the total organic carbon reserves in Chinese peatland. Thus, it accounts for the highest organic carbon accumulation of any peatland in China [14].

Unfortunately, due to climate warming, artificial drainage for pastures, and peat exploitation since the 1970s, Zoige peatland has suffered extensive biodiversity loss and ecosystem degradation, including severe peat deterioration [9]. The Zoige wetland has decreased by 30% in the past 30 years due to water table drawdown [15], and artificial drainage has been regarded to be the most important cause of Zoige wetland (including peatland) degradation [9]. Previous studies suggested that the carbon cycle in peatland could change rapidly with climate change [16]–[18] and that is sensitive to water table [4], [19]–[21]. Therefore, climate warming and a lowered of the water table could potentially create a carbon storage and ecosystem stability crisis in Zoige peatland.

Dissolved organic carbon (DOC) is the most active and sensitive indictor in the carbon cycle [22], and connects the biogeochemical cycle from terrestrial to aquatic ecosystems [23]. Hydrological losses of aquatic carbon can be of significant concern when determining carbon storage in peatlands [24] and may be increasing [25], [26]. Among the aquatic constituents of peatlands, DOC is generally considered to have the largest aquatic carbon flux [27], [28]. The peculiar water–peat interaction system and strong hydrological connectivity in peatlands ensures that the export of DOC from peatland to downstream plays a key role in the regional redistribution of terrestrial carbon [29] and the carbon balance [30]. Furthermore, the transfer of carbon from terrestrial peatland to fluvial downstream locations has a large influence on the water quality in aquatic ecosystems [31], [32]. Previous studies have warned that larger amounts of DOC feeding into downstream locations could increase the levels of aquatic organic acids, decrease the buffering ability of the water, and attenuate the penetration of visible and ultraviolet (UV) light due to changes in the water color [33]. This is likely to cause damage to the sustainable and stable development of aquatic ecosystems, such as their net primary productivity [34] and production of bacteria [2], [35]. A large body of literature has reported changes in the color or aromatic components of water in peatlands that has occurred in recent years [11], [31], [36], [37]. SUVA_254 nm_ was a useful parameter for determining the aromatic characteristic of DOC [38], and absorbance at 400 nm was used as a measure of the color composition [36] and could further indicate changes in DOC composition when combined with specific absorbance [39]. Therefore, DOC is likely to be an important part of the carbon cycle linking peatland and downstream ecosystems, although it is not the only pathway of carbon loss from an upland peatland.

The amount of DOC exported from peatlands is believed to depend on interactions between discharged water through peatland and the production and consumption of DOC within the peatland [4]. However, it has also been reported to increase with a higher discharge [25], [40], [41] without any effect on DOC concentration. Climate change can regulate the import and export of DOC [42], [43], mainly by controlling the most important environmental factors (i.e., temperature and the water table) affecting the peatland carbon cycle. A high water table and low soil temperatures are believed to be major reasons for the low decay rates, which could restrict the production of DOC compounds [44]–[46]. However, previous observations have indicated that DOC concentrations in peat could be either elevated [47]–[49] or lowered [4], [37], [50], [51] with a decline in the water level, which could be contributed to the complicated mechanisms and processes involved in the production, consumption, and transport of DOC in peat along with inevitable site-specific characteristics [52]. Similarly, high temperatures can not only improve DOC production through enhanced phenol oxidase activity but also increase the consumption of DOC [25], [43]. Thus, it is difficult to determine DOC concentrations in specific regions without performing practical experiments. Moreover, some studies have observed significant changes in water color and aromatic content with shifting water tables and soil temperatures at a range of sites [28], [36], [49], [52]–[54]. Many previous studies have produced inconsistent results regarding the effects of changes to the water table and/or warming on aquatic DOC release, with both factors able to impact DOC concentrations, the amount of discharge, or both, in a confounding way. Specifically, the response of these variables in peatlands could depend on the length of the observation period. For example, the response of DOC production to drought conditions in the year of drought may differ from that a few years after the drought [55], [56], and in a Tibetan alpine meadow experiment, the response of the aboveground environment to warming treatments in the third year was found to be different from the trend of the first two years [57]. Therefore, our observations in the year immediately after a controlled experiment are helpful for understanding how DOC export might react to climate change and anthropogenic drainage.

Zoige peatland is known to be undergoing a warming and drying climate trend [46], and severe artificial drainage [58]. Several studies in Zoige recently have reported that changes of temperature and/or water table could cause effect on the emissions of CH4 and CO2 [23], [26], [58], [59], and Luo et al. [60] has noticed that DOC could response to experimental warming and grazing. However, there is currently knowledge of the potential response of hydrological DOC loss to the variation of temperature and water table in Zoige peatland. Furthermore, most previous studies on DOC have been conducted countries other than China, particularly in Europe and North America. Therefore, investigating regarding the response of DOC export to warming and water table treatments could provide insight into the impact and mechanisms of climate warming and artificial drainage on the regional carbon budget of Zoige peatland, as well as provide guidance for the local protection and restoration of this deteriorating natural environment. Thus, we undertook a mesocosm experiment to investigate how the hydrological loss of DOC would respond to climatic warming and artificial drainage. The specific objectives were to determine whether the export quantity and concentration, as well as the qualities of DOC and the discharge of flow water, could respond significantly to water table and temperature manipulations. In terms of potential changes of DOC export, the study provides evidence of possible changes to the carbon cycle and storage under the impact of climate change or artificial disturbance and provides evidence for the need to protect and further restore the Zoige peatland.

## Methods

### Field Site

The peat columns used for mesocosms were collected from Zoige peatland in Hongyuan County, Sichuan Province, on the northeastern margin of the Qinghai–Tibet Plateau (32.76°N, 102.5°E), with a mean altitude of about 3,500 m. Peat was extracted in the area for energy production until 2003, which has left a peat layer of approximately 2 m deep and created severe long-term water shortages [61]. The vegetation community mainly consists of *Carex muliensis* (relative coverage of 41%) and *Kobresia setchwanensis* (39%), as well as a small number of scattered *Potentilla anserina* (15%) and *Plantago depressa* (11%). The topography and vegetation characteristics of the study area are shown in Figure S1. During the period 2002–2011, the site experienced a mean annual temperature and precipitation level of 2.27°C and 700 mm year^−1^, respectively. During that period, the mean temperature and precipitation from May to October were 8.26°C and 596.34 mm, respectively (data obtained from the China Meteorological Data Sharing Service System at http://cdc.cma.gov.cn/home.do). This study was conducted from May to October in 2012, when the mean temperature and precipitation were 8.65°C and 808 mm, respectively. Therefore, the site experienced higher rainfall and higher temperatures than the average of the previous 10 years.

The study was carried out on the private land of Mr. Jiang in Hongyuan County. Please contact the author first if further information is required. No further permits were required for the locations/activities in the study, and our work did not involve any endangered or protected species.

### Mesocosm Experiment

All peat columns were extracted intact from the source plot in December 2011, when the peat was totally frozen and easy to move and reset. Frozen peat cores (cuboid-shaped, with intact vegetation and peat structure) with a surface area of 1 m^2^ and a depth of 50–66 cm were carefully placed into stainless-steel barrels with only an open top. We used perforated stainless steel (diameter 9.0 cm) as a pocket sand filter (gravel particle size <4 mm), passing water through its inlet to maintain a near-natural infiltration rate. The perforated stainless-steel filter was buried into the peat column and connected by a drainage system to 5-L tanks in the closed bottom used to store the discharge [62]. The drainage system was connected to a manostat device with a similar pocket sand filter in the interface to lessen the peat outflow. Eighteen mesocosms were constructed for the manipulation of temperature and water table levels (three water table levels, two temperature, and three replicates, n = 18) in a crossed factorial experiment that commenced in May 2012. Positions of the water table level were controlled by hanger loops of the drainage system and set to 0 cm (W0), −10 cm (W1), and −20 cm (W2). They were calibrated using engraved rulers placed adjacent to the bottom of the steel barrels (i.e., the height of the water table was equal to the depth of the peat column plus the observed value). Warming treatment was achieved by using open top chambers (OTCs) during the snow-free period following Walker et al. [63], with 0.43-m-high polycarbonate solar panels placed outside of the mesocosms instead of infrared lamps. Actually, OTCs realize warming mainly through reducing both wind-speed and air convection and increasing incoming solar radiation [64], [65]. It can be confirmed by results of previous studies [63], [65], [66]. During the first growing season, we observed an overall temperature increase of 1.35°C (on an annual basis) was observed for the peat with a −10 cm water table in the warming mesocosms (T1) compared to the ambient mesocosms (T0) (Figure S2). The details on the experiment design in the study were shown in Figure S4.

To closely monitor the output–input water budget in the mesocosms, water discharged from the mesocosms, rainfall, and recharge water were measured using a gauge at least once a week, and more frequently for the first two measurements when rainfall occurred. Water in each mesocosm was mainly supplied by natural precipitation and supplemented by water pumped from a nearby drainage ditch to maintain the preset water table level when necessary. As the drainage ditch extended from the same continuous *C. muliensis* peatland, thus this supplementary water had a similar attributes to the water at the field site. We buried four HOBOPro data-loggers to record peat temperature at −10 cm depth in the mesocosms: two in warming mesocosms with a water table level of 0 cm and two in control mesocosms with a water table level of −20 cm. Monthly weather data from the Hongyuan County weather station were collected for reference.

### Sample Analysis

During the study period, discharged water was collected every month for DOC analysis during the growing season (May–October) in 2012. Water samples collected from the manostat tanks were mixed well before sampling, stored in sterile containers (volume 100 ml), and then filtrated through a syringe microfilter (0.45 µm) as preparation for further testing. The DOC concentration was equivalent to total carbon (TC) minus dissolved inorganic carbon (DIC), and both were determined directly using a TOC/TN analyzer (Multi N/C3100TOC/TN; Analytik Jena, Germany). TC was measured by wet combustion, and DIC was measured after sample acidification by 10% H_3_PO_4_ as proposed by Guo et al. [67]. The water budget data and the measured DOC concentrations were used to estimate DOC export by Method 3 proposed by Walling and Webb [68]. The UV absorbances of filtered water samples at wavelengths of 254 nm and 400 nm were determined using a UV-visible spectrophotometer (UV-2600; Shimadzu, Kyoto, Japan). UV absorption characteristics of DOC are generally measured to obtain information regarding changes in the composition of DOC compounds. We thus determined the characteristics of DOC composition by means of four related measurements of specific- and UV absorption (i.e., Abs_254 nm_, SUVA_254 nm_, Abs_400 nm_, and SUVA_400 nm_).

### Statistical Analysis

Statistical analysis was done using a three-way ANOVA, including the effect of interactions between the time variable (month) and the two treatments on the monthly changes of DOC. Then a sequential full model of two-way repeated-measures ANOVA and main effect analysis and a Sidak post hoc comparison of means test were successively conducted to determine the effects of two treatments. All of these analyses were conducted after testing for essential homogeneity of variance (*p*>0.05, meaning that variances were homogenous; see Table. 1). Further correlation and regression analyses were conducted to determine the relationships of the monthly mean DOC concentrations and DOC export values with the corresponding peat temperature and precipitation. We also conducted linear regression analysis to determine what proportion of the two treatments and discharged volumes respectively. All statistical analyses were performed using SPSS 17.0 (SPSS Inc., Chicago, IL, USA).

**Table 1:** *P*-values of a two-way ANOVA and Levene's test for the effects of the water table level, temperature, and their interactions on the amount of annual DOC export, DOC concentration, absorbance and specific absorbance, and water discharge.

Treatment	DOC		Discharge	Absorbances and specific absorbances			
	Export	Concentration	Discharge	Abs_254 nm_	SUVA_254_	Abs_400 nm_	SUVA_400_
Water table	<0.001**	<0.001**	0.037*	0.001**	0.005**	0.003**	0.008**
Temperature	0.075	0.012**	0.764	0.007**	0.008**	0.010**	0.018**
Water table × Temperature	0.734	0.735	0.553	0.689	0.077	0.439	0.431
Levene's Test	0.179	0.318	0.235	0.520	0.318	0.616	0.110

*indicates a significant difference (*p*<0.05, n = 18)

**indicates a highly significant difference (*p*<0.01, n = 18).

## Results

### Microclimate in Mesocosms and Its Correlation with DOC

#### Soil Temperature in Peat and Precipitation

The mean monthly temperatures in the mesocosms showed that peat temperature (from May to October) was significantly higher on average in warmed (11.95 ± 4.03°C) than in the controlled mesocosms (10.60 ± 4.25°C, *p = *0.003; n* = *164; Figure S2). The mean monthly precipitation measured with the rain gauge in the mesocosms was 139.14 mm (139.14 L m^−2^) during the growing season, which was very similar to the value of 134.92 mm recorded by the meteorological station in Hongyuan County. In general, precipitation in all mesocosms was nearly identical regardless of the possible changes in discharge and evapotranspiration among mesocosms located at the same site. A mean number of 17.4 rainy days per month occurred from May to September. Specifically, October only had five rainy days, and June and July each had twenty-one rainy days. The average discharge in the mesocosms was 281.19 L m^−2^ year^−1^, which accounted for 32% of the water input (precipitation and water recharge) and 34% of the rainfall. In contrast, the annual precipitation of temperate biomes (e.g. temperate forest) in China ranges from 400 to 650 mm, i.e., less than the average precipitation (834.84 mm for 6 months) at Zoige. This suggests an abundance of precipitation in Zoige peatland, which is necessary to maintain its year-round spongy condition. It was found that the effect of warming on discharge was nonsignificant in the experiment (*p>*0.05; Table 1). Meanwhile, the interactive effect of warming and water table on discharge was also insignificant (*p>*0.05; Table 1).

#### Correlation of Peat Temperature and Precipitation with DOC

One year study provides limited perspective on the interannual patterns of DOC hydrological export. However, we obtained extra information by the correlation analysis between the two main microclimatic factors and the mean monthly concentration and export of DOC, see Figure S3. Monthly DOC export was positively correlated with peat temperature (*R* ^2^ = 0.4984, *p<*0.01; n* = *24) and precipitation (*R* ^2^ = 0.8982, *p<*0.01; n* = *6), while the mean monthly DOC concentration was significantly correlated with peat temperature (*R* ^2^ = 0.4025, *p<*0.01; n* = *24), but had a nonsignificant correlation with precipitation (*R* ^2^ = 0.3046, *p = *0.128; n* = *6).

### Annual Export of DOC

Neither the water table × warming nor the month × two controlling-factor interactions were statistically significant. Thus, we examined the single effect of water table and temperature manipulation on the measured variables in the study (i.e. DOC export, concentration, and quality, and the water budget).

**Figure 1:**
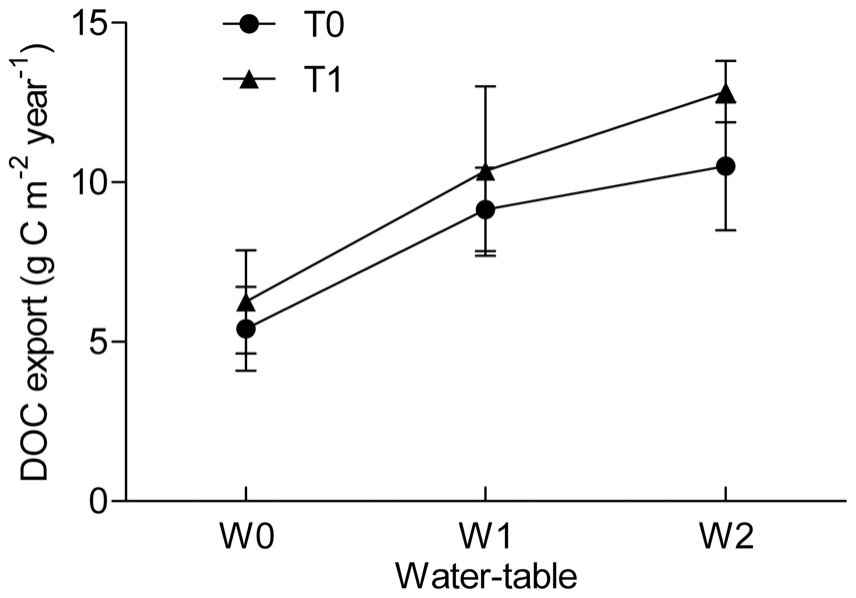
Effect of water table levels and temperature on DOC annual export. Data are means ± standard error. T0 and T1 correspond to ambient temperature and warming temperature, respectively, and W0, W1, and W2 indicate water table depths of 0 cm, −10 cm, and −20 cm, respectively.

We found a difference between the experimental warming treatment and water table treatment in terms of the annual amount of DOC exported. During the period of the study, the manipulation of water table depth in the mesocosms significantly influenced DOC export (*p<*0.001; Figure 1). The export of DOC displayed an upward trend with decreasing water levels. DOC export was 5.76 ± 0.63 g C m^−2^ year^−1^ when the water table was at 0 cm, significantly lower than the levels of 9.75 ± 0.84 g C m^−2^ year^−1^ (−10 cm water-level; *p = *0.004) and 11.65 ± 1.68 g C m^−2^ year^−1^ (−20 cm water-level; *p<*0.001) respectively. Although all values were within the range (5–40 C m^−2^ year^−1^) found in the natural peatland [3], the results indicated that DOC export would increase by 69% and 102% annually if the water table at 0 cm was lowered by 10 cm and 20 cm, respectively. In contrast, no significant effect on DOC export was observed in the warming treatments when the peat temperature was raised by 1.35°C (9.81±3.32 g C m^−2^ year^−1^ vs. 8.30±1.82 g C m^−2^ year^−1^, *p = *0.059) throughout the growing season. Previous studies have demonstrated that DOC loss during the nongrowing season is similar between controlled and manipulated sites [41]. Therefore, our estimation of DOC export within the growing season did not suggest an alteration in its overall tendency throughout the year.

**Figure 2:**
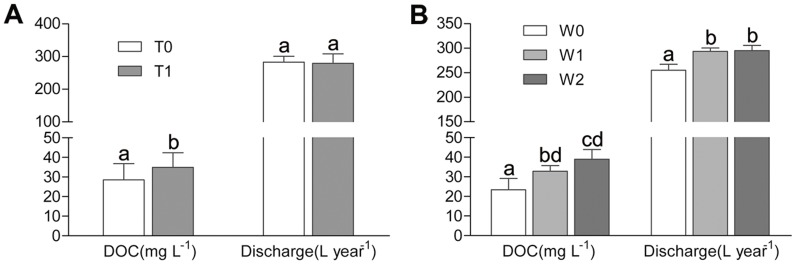
Variations in DOC concentrations and discharge volumes under different treatments. Data are means ± standard error. Same letter superscripts denote insignificant differences among the three water table levels from post hoc tests.

Eighty-seven percent of the variability in DOC annual export was explained by the combination of the water table level, temperature, and discharge. Among these variables, the level of the water table was the most important predictor, explaining more than 68% of the variation in DOC annual export. Furthermore, discharge was found to be significantly affected only by the water table treatment (*p = *0.028, n* = *18; Figure 2). The discharge volume was 265.08 ± 1.88 L year^−1^ when the water table was at 0 cm, slightly lower than 293.58.04 ± 6.71 L year^−1^ (−10 cm water-level; *p = *0.063) and significantly lower than 294.92 ± 10.84 L year^−1^ (−20 cm water-level; *p = *0.050). In contrast, the discharge in the warming treatment was nonsignificantly smaller (283.11 ± 17.66 L vs. 279.28 ± 29.28 L). Correlation analysis also showed that water table levels were significantly negatively correlated with discharge (*R* ^2^ = 0.31, *p = *0.008; n* = *18), while experimental warming was almost irrelevant to discharge (*R* ^2^ = 0.004, *p = *0.398; n* = *18).

### DOC Concentrations

DOC concentrations in discharged water varied significantly between the warmed and ambient temperature treatments as well as among the three water table treatments, despite the differences in their effects on DOC export. As shown in Figure 2, the DOC concentration was lower (23.18±5.76 mg L^−1^) when the position of the water table level was at 0 cm, significantly lower than 32.81±2.88 mg L^−1^ (−10 cm water-level; *p = *0.028) and 38.92±4.98 mg L^−1^ (−20 cm water-level; *p = *0.001). Similarly, DOC concentration was higher in the warmed mesocosms (34.90±5.25 mg L^−1^, *p = *0.005) than in the ambient temperature mesocosms (28.48±8.13 mg L^−1^). In addition, almost 78% of the variation in DOC concentrations was explained by water table and temperature treatments, and 61% of these could be contributed to the water table treatment alone.

### Qualities of DOC

**Figure 3:**
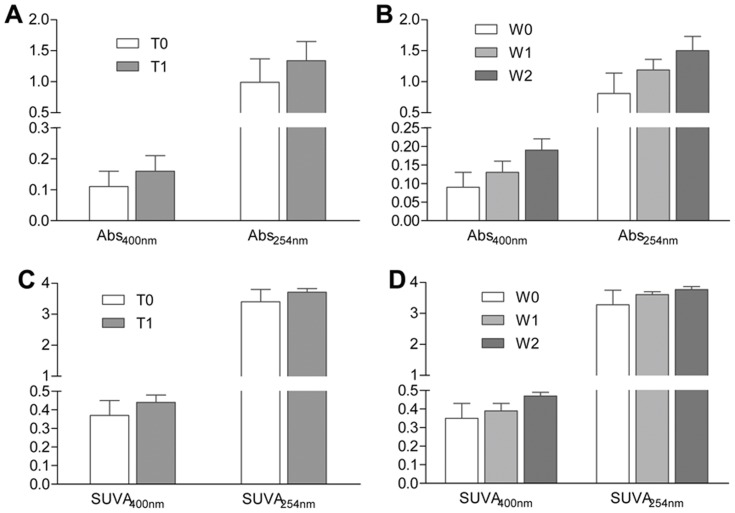
Effects of water table levels and temperature treatments on Abs_254 nm_, Abs_400 nm_, SUVA_254 nm_, and SUVA_400 nm_. Data are means ± standard error. Same letter superscripts denote nonsignificant differences among the three water table levels from post hoc tests.

There were similar trends for the effects of experimental warming and water table level on absorbance (at wavelengths of 254 nm and 400 nm) and specific absorbance (SUVA_254 nm_ and SUVA_400 nm_) of DOC in the filtered discharge. These four measures of the quality of DOC were all significantly higher under the warming treatment (*p = *0.004, *p = *0.016, *p = *0.004, and *p = *0.015, respectively; Table 1, Figure 3) than in the control. Similarly, values of the four measures for a lower water table were significantly higher than those observed at higher water table level (*p<*0.001, *p = *0.010, *p<*0.001, *p = *0.006, respectively; Table 1). We assessed the impact of the three positions of the water table level on the four DOC quality measures using a multiple comparison analysis as shown in Figure 3. Therefore, the results above showed that the water table and warming treatments clearly led to several changes in both the quality and absolute DOC concentrations of DOC, indicating a higher aromatic content and changes in the color of downstream water.

## Discussion

### Effect of Water Table Treatment on Export, Concentration, and Qualities of DOC

Water table manipulation had significant effects on the annual amount of DOC exported, DOC concentration, and the water discharge. Lower water tables were often accompanied by higher DOC exports and concentrations, and a larger discharge volume. These effects were significant when the water table at 0 cm was lowered to −10 cm and −20 cm. This supports the observed variation in the export of DOC, possibly due to site-specific characteristics, and is mainly derived from both fluctuations in the DOC concentration in runoff water and the quantity of water discharged, which disagrees with several previous studies [4], [26], [69]. Peatland has its own specific features such as plant community construction [70] and hydro-topographical characteristics [67]. Meanwhile, the export of DOC varies with catchment properties and hydrogeologic setting [71], such as precipitation, evapotranspiration [72] and annual runoff [73]. Therefore, it may have disparate performances in DOC production and export which may result in difficulty to draw a universal conclusion [67]. Actually, most reported studies on the response of DOC to water table were done in Europe [74] or North America [4], [69]. In this study, when the water table at 0 cm was artificially lowered to −10 cm and −20 cm, DOC export increased by 69% and 102%, respectively. This suggests that an estimated extra 18.4 × 10^9^ g C and 27.2 × 10^9^ g C, respectively, would be transported downstream during the growing season in Zoige. Our ranges of estimated DOC exports with a water table level of −10 cm and −20 cm (9.75–11.65 g C m^−2^ year^−1^) were similar to those (8.4–11.3 C m^−2^ year^−1^) observed in Quebec, Canada, in two growing seasons following a water table drawdown [41]. This could result in a substantial DOC loading into downstream ecosystems, potentially altering the physical and chemical characteristics of aquatic ecosystems, such as acidity, light penetration, and metal and nutrient availability [75]. Such indirect effects on aquatic ecosystems may be the most severe consequence of the elevated DOC export from peatlands. Increases in DOC export may also indicate shifts in the carbon budget, suggesting either a decrease in carbon uptake and storage, or an increase in the turnover of organic carbon [39].

As expected, DOC concentrations were elevated when the water level declined, which agreed with the results of several previous studies [47]–[49], but disagreed with some other published observations [4], [37], [50], [51]. These inconsistencies reflect the complicated mechanisms and processes involved in the production, consumption, and transport of DOC in peat [52]. Initially, water table drawdown, which is closely associated with peat moisture content, can promote aerobic respiration [76], [77] and release the activity of degrading hydrolase enzymes in peat, which is supported by reported changes in the specific absorbance of DOC [78]. Biomass is often related to water table, larger biomass being associated with lower water table [79]. Besides, water table could also affect decomposition indirectly through changes in plant community composition [80] or reduce productivity and even cause death in wetland species as the water table is lowered too far [79], thus it can't be determined water table effects on decomposition through changes in plant community or microbial activity within the results of our study. These two mechanisms described above lead to an increase in DOC production, which can then be flushed out from stagnant peat horizons [41] during rainfall. Furthermore, studies have shown that this could lead to peat subsidence and a lower porosity when the water level declines [42], [81], likely resulting in slower interflow and a longer residence time for water moving through peat. This may also contribute to more DOC compounds from peat transferring into flowing water. There were 17.4 rainy days every month from May to September 2012 in Zoige, with a maximum of 21 in each of June and July. According to Harrison et al. [82], this high rainfall rate may have potential effect on DOC concentration in water by promoting DOC release from peat soil. Moreover, the water discharge also increased when the level of the water table was lowered in the mesocosm experiment. This suggests a decreased capacity for water storage, which might exacerbate severe water loss and peat erosion if artificial water drainage continues in Zoige peatland. Higher DOC concentrations could also create problems for the stable and sustainable development of peatland ecosystems because it would alter aquatic habitats through its effect on pH and various biological activities (e.g., transportation of nutrients). The weak correlation between precipitation and DOC concentration also indicates that precipitation could promote the export of DOC with a limited enhancement of the DOC concentration. It also indicates that the elevated DOC in runoff water was the main contributor to the high discharge rather than the high concentration observed under higher rainfall.

The changes in DOC concentrations resulting from the water table treatment were accompanied by changes in UV absorbance characteristics. Our results showed that the aromatic and colored components of DOC increased when the water table was low, suggesting a possible increase in peat degradation. The colored components of DOC, as measured by absorbance at 400 nm and SUVA_400 nm_, increased sharply when the water table at 0 cm level was lowered to −20 cm, but showed only a slight decline when it was lowered to −10 cm. The increase in DOC aromaticity, measured by absorbance at 254 nm and SUVA_254 nm_, implied that more aromatic DOC substances should occur in discharge water following a decline in the water table level. Recent studies of Zoige peatland have demonstrated that aromaticity has substantially increased in sites that have experienced a long period of aerobic oxidation and water loss [11]. This is in accordance with our observation of a higher aromatic content when the water table was lower because more peat would be exposed to the air, resulting in increased aerobic respiration [76]. This would also support a reduction in the colored components and aromatic content when the water table rises, which has also been observed elsewhere [36], [49], . Consequently, DOC in runoff consists of more colored and aromatic components when the water table was drawdown, making it less accessible to microbes within the fluvial ecosystem [54]. Finally, as our results suggested, it would lead to carrying more DOC compounds downstream.

### Effect of Warming on Export, Concentration, and Qualities of DOC

Experimental warming at a rate of 1.35°C·year^−1^ in peat had a significant effect on DOC concentration but limited effects on DOC export and discharge. DOC concentrations in warming mesocosms were higher than in normal mesocosms. However, the observed variability of DOC concentrations generally had a limited impact on the hydrological export of DOC which is explained by the relevance of the discharge volume to DOC exports and displayed a nonsignificant correlation with warming in our experiment. Some studies have observed high DOC concentrations at higher temperatures that increased the amount of DOC exported for as long as 12 years under natural warming conditions [25], whereas others have reported a decrease in DOC exports due to the lower discharge following a temperature increase of 1.6–4.1°C [4]. In the study, warming mesocosms have significantly higher DOC concentration but with insignificant lower discharge, which may result from its specific feature (such as evapotranspiration) [72] in Zoige that differs from anywhere else. Therefore, we assumed that temperature variation may have a complex influence on DOC export that is probably associated with both the rate of warming and the temporal scale. It is important to consider both present and future climate change when investigating the effects of experimental warming on peatland carbon turnover.

Temperature is the main factor influencing bacterial metabolism and the rate of decomposition of organic materials, and it also affects DOC dynamics in ecosystems [83]. High temperatures can not increase DOC production through enhanced phenol oxidase activity, but it also can increase the consumption of DOC [25], [43]. Thus, determining the DOC concentration in specific regions is difficult without conducting practical experiments. Warming can decrease plant species richness but increase aboveground net primary production [84], thus it may influence inputs of carbon into peatland and lead to unstable DOC concentrations. Our correlation analysis in our experiment indicated that the monthly mean DOC concentration in the discharge increased with temperature. This suggests that the higher temperature increased the DOC concentration because the enhanced decomposition exceeded the gain in DOC consumption in the first year following a rise in temperature. Walker et al. [63] suggested that warming in OTC experiments increased the height and cover of deciduous shrubs and graminoids and decreased species diversity and evenness, implying that the increased DOC concentration we observed might also be attributable to the changes in plant primary productivity. However, temperature had a weak effect on the water discharge during the whole growing season, indicating a relatively stable discharge volume independent of the warming treatment, and differing slightly from the observations of Bridgham et al. [62]. However, this is understandable given the relatively plentiful precipitation at the study site as well as the relatively small change in peat temperature.

Similarly, all of the colored components displayed an upward trend with warming in the study, which was contrary to the results obtained in a laboratory experiment by Tang et al. [52]. But one recent study showed that warming could cause a shift in the composition of bacterial communities in the surface (1–3 cm) and middle layers (9–11 cm) of peat [85], which supported our observations of greater colored and aromatic content under warmer conditions. Therefore, our results indicated that the rising temperature could influence the composition of peat (especially color and aromatic content). Furthermore, peat degradation might arise following climatic warming according to the results, probably due to potential shifts in the function and structure of microbial communities in the peatland, a hypothesis that requires further investigation.

## Conclusion

We investigated the response of the hydrological export of DOC in Zoige peatland to changes in the water table level and temperature. Our one-year study provides a basis for understanding the rapid response of the carbon budget in Zoige peatland to climate change and/or artificial drainage, as well as the potential damage to downstream ecosystems, particularly in the Yellow River.

The differences between the water table and peat-temperature treatments implied that future short-duration water table drawdown events could have a greater impact than rising temperature on the export of DOC. In this study, water table effects DOC concentration and export as well as discharge, while temperature treatment only causes obvious effect on DOC concentration. It probably derives from that water table drawdown influenced temperature patterns in the decomposing litter [80] in the crossed factorial experiment. Meanwhile, the experimental warming may also not be high enough for changing the amount of DOC export, as warming could also increase evapotranspiration and therefore decrease discharge [62], which can be known from the relatively smaller discharge in warming mesocosm in the study. Thus, it supported the view that the influence of the water table or water content on peatland ecosystems (such as DOC loss in the study) is stronger than in variations in other environmental conditions, such as temperature [79]. The two experimental water table positions (−10 m and −20 m) resulted in increases in the annual export of DOC by 69% and 102%, respectively, through both a higher DOC concentration and larger discharge volume, indicating the potential release of both carbon and water from peatland after the level of the water table is lowered. The temperature treatment resulted in clear changes in the DOC concentration but had a limited effect on DOC export, probably indicating a shift in the turnover rate of organic carbon in peat because temperature is the main factor affecting bacterial metabolism and the rate of decomposition of organic materials. The nonsignificant effect of warming on DOC export and the notably positive relationship between mean monthly DOC export and peat temperature, which resulted from the shortage of recorded data for all mesocosms, appear contradictory, and suggest that further studies should be careful to consider these issues. Variable water levels and temperatures changed the absorbances of DOC in the year immediately after this experiment. This result suggests the varied nature or qualities of DOC that might influence fluvial systems, and also warns that using absorbance records as a proxy for DOC concentrations when studied in peatland should be done with caution.

Therefore, our observations in the first year immediately after the controlled experiment were helpful for understanding how the carbon budget might react to climate change and anthropogenic interference (i.e., drainage). This mesocosm experiment also provides useful information for local protection and sustainable development in Zoige peatland. Additional experiments and observations are required to achieve a comprehensive understanding of the carbon cycle (such as DOC fluxes) in peatlands facing changing environmental conditions.

## Supporting Information

Figure S1 Topography and vegetation characteristics of the study area. (A) Drainage ditch. (B) Vegetation community growing in shallow water. (C) Scattered vegetation surrounded by surface water. All photographs were taken during May 2012 in Hongyuan County located in Zoige peatland.
(TIF)

Figure S2 Peat temperature at −10 cm depth and precipitation during the growing season in 2012.
(TIF)

Figure S3 Correlation analysis of temperature and precipitation with DOC. (A) Results of the correlation analysis between peat temperature recorded in four mesocosms and corresponding mean monthly export and concentration of DOC (DOC concentration: y = 1.4573x +9.9416, *R* ^2^ = 0.4025, *p<*0.01, n* = *24; DOC export: y = 0.2043x −0.9177, *R* ^2^ = 0.4984, *p<*0.01, n* = *24). (B) Results of the correlation analysis between mean monthly precipitation and mean monthly export and concentration of DOC in all mesocosms (DOC concentration: y = 0.0379x +26.409, *R* ^2^ = 0.3046, *p = *0.128, n* = *6; DOC export: y = 0.013x −0.2987, *R* ^2^ = 0.8982, *p<*0.01, n* = *6).
(TIF)

Figure S4 The schematic drawing of the mesocosm in the study. The references of the number are shown as below: 1. Polycarbonate solar panels; 2. Water intake system; 3. Drainage system; 4. Water storage barrel; 201. Observation tube of water-level; 202. Sand filter pocket of inlet; 301. Sand filter pocket of outlet; 302. High pressure valves; 303. Water pipe; 304. Hanger loop; 305. Observation rule.
(TIF)

## References

[1] Aselmann I, Crutzen PJ (1989) Global distribution of natural fresh-water wetlands and rice paddies, their net primary productivity seasonality and possible methane emissions. Journal of Atmospheric Chemistry 8: 307–358.

[2] Hobbie JE (1992) Microbial control of dissolved organic-carbon in lakes - research for the Future. Hydrobiologia 229: 169–180.

[3] Moore TR, Roulet NT, Waddington JM (1998) Uncertainty in predicting the effect of climatic change on the carbon cycling of Canadian peatlands. Climatic Change 40: 229–245.

[4] Pastor J, Solin J, Bridgham SD, Updegraff K, Harth C, et al (2003) Global warming and the export of dissolved organic carbon from boreal peatlands. Oikos 100: 380–386.

[5] Turunen J (2008) Development of Finnish peatland area and carbon storage 1950–2000. Helsinski, FINLANDE: Finnish Environment Institute. 16 p.

[6] Yu Z, Beilman DW, Jones MC (2013) Sensitivity of northern peatland carbon dynamics to Holocene climate change. Carbon Cycling in Northern Peatlands: American Geophysical Union. pp. 55–69.

[7] Moore TR, Dalva M (1993) The influence of temperature and water table position on carbon dioxide and methane emissions from laboratory columns of peatland soils. Journal of Soil Science 44: 651–664.

[8] Price JS (2003) Role and character of seasonal peat soil deformation on the hydrology of undisturbed and cutover peatlands. Water Resources Research 39: 1241.

[9] Xiang S, Guo R, Wu N, Sun S (2009) Current status and future prospects of Zoige Marsh in Eastern Qinghai-Tibet Plateau. Ecological Engineering 35: 553–562.

[10] Shi C-c, Tu J (2009) Remote Sensing Monitory Study on Land Desertification in Ruoergai Plateau of Sichuan Province during 40 Years. Southwest China Journal of Agricultural Sciences 6: 035 (in Chinese)..

[11] Guo X, Du W, Wang X, Yang Z (2013) Degradation and structure change of humic acids corresponding to water decline in Zoige peatland, Qinghai-Tibet Plateau. Sci Total Environ 445–446: 231–236. 10.1016/j.scitotenv.2012.12.048 23334317

[12] Zhang X, Lv X, Gu H (2005) To analyze threats, to describe present conservation situation and to provide management advices of the Ruoergai marshes. Wetland Sci 3: 292–297 (in Chinese)..

[13] SAFS (Sichuan Academy of Forest Science) (2006) Scientific investigation report on Zoige marsh. Chengdu: Sichuan Science and Technology Press(in Chinese).

[14] Wang M, Liu Z, Ma X, Wang G (2012) Division of organic carbon reserves of peatlands in China. Wetland Sci 10: 156–163 (in Chinese)..

[15] Gao J (2006) Degradation factor analysis and solutions of Ruoergai Wetland in Sichuan. Sichuan Environ 25: 48–53 (in Chinese)..

[16] Gorham E (1991) Northern peatlands- role in the carbon-cycle and probable responses to climatic warming. Ecological Applications 1: 182–195. 27755660 10.2307/1941811

[17] Trettin CC, Laiho R, Minkkinen K, Laine J (2006) Influence of climate change factors on carbon dynamics in northern forested peatlands. Canadian Journal of Soil Science 86: 269–280.

[18] Bridgham SD, Pastor J, Dewey B, Weltzin JF, Updegraff K (2008) Rapid carbon response of peatlands to climate change. Ecology 89: 3041–3048. 10.1890/08-0279.1 31766807

[19] Hogg EH, Lieffers VJ, Wein RW (1992) Potential carbon losses from peat profiles - effects of temperature, drought cycles, and fire. Ecological Applications 2: 298–306. 27759264 10.2307/1941863

[20] Freeman C, Lock MA, Reynolds B (1993) Fluxes of CO2, CH4 and N2O from a Welsh peatland following simulation of water-table draw-down - potential feedback to climatic-change. Biogeochemistry 19: 51–60.

[21] Bohrer G, Chen H, Wu N, Wang Y, Zhu D, et al (2013) Inter-Annual Variations of Methane Emission from an Open Fen on the Qinghai-Tibetan Plateau: A Three-Year Study. PLoS ONE 8: e53878. 23342029 10.1371/journal.pone.0053878 PMC3544678

[22] Evans CD, Chapman PJ, Clark JM, Monteith DT, Cresser MS (2006) Alternative explanations for rising dissolved organic carbon export from organic soils. Global Change Biology 12: 2044–2053.

[23] Zhang G, Tian J, Jiang NA, Guo X, Wang Y, et al (2008) Methanogen community in Zoige wetland of Tibetan plateau and phenotypic characterization of a dominant uncultured methanogen cluster ZC-I. Environmental microbiology 10: 1850–1860. 18373675 10.1111/j.1462-2920.2008.01606.x

[24] Billett MF, Palmer SM, Hope D, Deacon C, Storeton-West R, et al (2004) Linking land-atmosphere-stream carbon fluxes in a lowland peatland system. Global Biogeochemical Cycles 18: GB1024.

[25] Freeman C, Evans CD, Monteith DT, Reynolds B, Fenner N (2001) Export of organic carbon from peat soils. Nature 412: 785–785. 10.1038/35090628 11518954

[26] Chen H, Yao S, Wu N, Wang Y, Luo P, et al (2008) Determinants influencing seasonal variations of methane emissions from alpine wetlands in Zoige Plateau and their implications. Journal of Geophysical Research: Atmospheres 113: D12303.

[27] Limpens J, Berendse F, Blodau C, Canadell JG, Freeman C, et al (2008) Peatlands and the carbon cycle: from local processes to global implications – a synthesis. Biogeosciences 5: 1475–1491.

[28] Dinsmore KJ, Billett MF, Dyson KE (2013) Temperature and precipitation drive temporal variability in aquatic carbon and GHG concentrations and fluxes in a peatland catchment. Global Change Biology 19: 2133–2148. 23568485 10.1111/gcb.12209

[29] Aerts R, De Caluwe H (1999) Nitrogen deposition effects on carbon dioxide and methane emissions from temperate peatland soils. Oikos 84: 44–54.

[30] Arnosti C, Holmer M (2003) Carbon cycling in a continental margin sediment: contrasts between organic matter characteristics and remineralization rates and pathways. Estuarine Coastal and Shelf Science 58: 197–208.

[31] Wallage ZE, Holden J (2010) Spatial and temporal variability in the relationship between water colour and dissolved organic carbon in blanket peat pore waters. Science of The Total Environment 408: 6235–6242. 20888621 10.1016/j.scitotenv.2010.09.009

[32] Chin W-C, Lennon JT, Hamilton SK, Muscarella ME, Grandy AS, et al (2013) A Source of Terrestrial Organic Carbon to Investigate the Browning of Aquatic Ecosystems. PLoS ONE 8: e75771. 24124511 10.1371/journal.pone.0075771 PMC3790824

[33] Evans CD, Monteith DT, Cooper DM (2005) Long-term increases in surface water dissolved organic carbon: Observations, possible causes and environmental impacts. Environmental Pollution 137: 55–71. 15944040 10.1016/j.envpol.2004.12.031

[34] Carpenter SR, Pace ML (1997) Dystrophy and eutrophy in lake ecosystems: Implications of fluctuating inputs. Oikos 78: 3–14.

[35] Wetzel RG (1992) Gradient-dominated ecosystems - sources and regulatory functions of dissolved organic-matter in fresh-water ecosystems. Hydrobiologia 229: 181–198.

[36] Wallage ZE, Holden J, McDonald AT (2006) Drain blocking: An effective treatment for reducing dissolved organic carbon loss and water discolouration in a drained peatland. Science of the Total Environment 367: 811–821. 16600338 10.1016/j.scitotenv.2006.02.010

[37] Grayson R, Holden J (2012) Continuous measurement of spectrophotometric absorbance in peatland streamwater in northern England: implications for understanding fluvial carbon fluxes. Hydrological Processes 26: 27–39.

[38] Weishaar JL, Aiken GR, Bergamaschi BA, Fram MS, Fujii R, et al (2003) Evaluation of specific ultraviolet absorbance as an indicator of the chemical composition and reactivity of dissolved organic carbon. Environmental Science & Technology 37: 4702–4708. 14594381 10.1021/es030360x

[39] Worrall F, Armstrong A, Adamson JK (2007) The effects of burning and sheep-grazing on water table depth and soil water quality in a upland peat. Journal of Hydrology 339: 1–14.

[40] Fraser CJD, Roulet NT, Moore TR (2001) Hydrology and dissolved organic carbon biogeochemistry in an ombrotrophic bog. Hydrological Processes 15: 3151–3166.

[41] Strack M, Waddington JM, Bourbonniere RA, Buckton EL, Shaw K, et al (2008) Effect of water table drawdown on peatland dissolved organic carbon export and dynamics. Hydrological Processes 22: 3373–3385.

[42] Sommer M (2006) Influence of soil pattern on matter transport in and from terrestrial biogeosystems—A new concept for landscape pedology. Geoderma 133: 107–123.

[43] Briggs J, Large DJ, Snape C, Drage T, Whittles D, et al (2007) Influence of climate and hydrology on carbon in an early Miocene peatland. Earth and Planetary Science Letters 253: 445–454.

[44] Strack M, Waddington JM, Tuittila ES (2004) Effect of water table drawdown on northern peatland methane dynamics: Implications for climate change. Global Biogeochemical Cycles 18: GB4003.

[45] Scanlon D, Moore T (2000) Carbon dioxide production from peatland soil profiles: The influence of temperature, oxic/anoxic conditions and substrate. Soil Science 165: 153–160.

[46] Morris PJ, Belyea LR, Baird AJ (2011) Ecohydrological feedbacks in peatland development: A theoretical modelling study. Journal of Ecology 99: 1190–1201.

[47] Dai Y, Luo Y, Wang C, Shen Y, Ma Z, et al (2010) Climate variation and abrupt change in wetland of Zoig Plateau during 1961 and 2008. Journal of Glaciology and Geocryology 32: 35–42 (in Chinese)..

[48] Jager DF, Wilmking M, Kukkonen JVK (2009) The influence of summer seasonal extremes on dissolved organic carbon export from a boreal peatland catchment: Evidence from one dry and one wet growing season. Science of The Total Environment 407: 1373–1382. 18977515 10.1016/j.scitotenv.2008.10.005

[49] Blodau C, Siems M (2012) Drainage-induced forest growth alters belowground carbon biogeochemistry in the Mer Bleue bog, Canada. Biogeochemistry 107: 107–123.

[50] Sapek A, Sapek B, Chrzanowski S, Urbaniak M (2009) Nutrient mobilisation and losses related to the groundwater level in low peat soils. International Journal of Environment and Pollution 37: 398–408.

[51] Ellis T, Hill PW, Fenner N, Williams GG, Godbold D, et al (2009) The interactive effects of elevated carbon dioxide and water table draw-down on carbon cycling in a Welsh ombrotrophic bog. Ecological Engineering 35: 978–986.

[52] Tang R, Clark JM, Bond T, Graham N, Hughes D, et al (2013) Assessment of potential climate change impacts on peatland dissolved organic carbon release and drinking water treatment from laboratory experiments. Environmental Pollution 173: 270–277. 23207497 10.1016/j.envpol.2012.09.022

[53] Watts CD, Naden PS, Machell J, Banks J (2001) Long term variation in water colour from Yorkshire catchments. Science of The Total Environment 278: 57–72. 11669277 10.1016/s0048-9697(00)00888-3

[54] Wilson L, Wilson J, Holden J, Johnstone I, Armstrong A, et al (2011) Ditch blocking, water chemistry and organic carbon flux: Evidence that blanket bog restoration reduces erosion and fluvial carbon loss. Science of the Total Environment 409: 2010–2018. 21440287 10.1016/j.scitotenv.2011.02.036

[55] Mitchell GN (1990) Natural discoloration of freshwater: Chemical composition and environmental genesis. Progress in Physical Geography 14: 317–334.

[56] Mitchell G, McDonald AT (1992) Discolouration of water by peat following induced drought and rainfall simulation. Water Research 26: 321–326.

[57] Li G, Liu Y, Frelich LE, Sun S (2011) Experimental warming induces degradation of a Tibetan alpine meadow through trophic interactions. Journal of Applied Ecology 48: 659–667.

[58] Chen H, Wu N, Wang Y, Zhu D, Zhu Qa, et al (2013) Inter-Annual Variations of Methane Emission from an Open Fen on the Qinghai-Tibetan Plateau: A Three-Year Study. PLoS ONE 8: e53878. 23342029 10.1371/journal.pone.0053878 PMC3544678

[59] Yanbin H, Yanfen W, Xurong M, Xiangzhong H, Xiaoyong C, et al (2008) CO2H2O and energy exchange of an Inner Mongolia steppe ecosystem during a dry and wet year. Acta Oecologica-international Journal Of Ecology 33: 133–143.

[60] Luo C, Xu G, Wang Y, Wang S, Lin X, et al (2009) Effects of grazing and experimental warming on DOC concentrations in the soil solution on the Qinghai-Tibet plateau. Soil Biology and Biochemistry 41: 2493–2500.

[61] Zhang XH, Liu HY, Baker C, Graham S (2012) Restoration approaches used for degraded peatlands in Ruoergai (Zoige), Tibetan Plateau, China, for sustainable land management. Ecological Engineering 38: 86–92.

[62] Bridgham SD, Pastor J, Updegraff K, Malterer TJ, Johnson K, et al (1999) Ecosystem control over temperature and energy flux in Northern peatlands. Ecological Applications 9: 1345–1358.

[63] Walker MD, Wahren CH, Hollister RD, Henry GH, Ahlquist LE, et al (2006) Plant community responses to experimental warming across the tundra biome. Proc Natl Acad Sci U S A 103: 1342–1346. 16428292 10.1073/pnas.0503198103 PMC1360515

[64] Debevec EM, MacLean JrSF (1993) Design of greenhouses for the manipulation of temperature in tundra plant communities. Arctic and Alpine Research: 56–62.

[65] Turetsky M, Treat C, Waldrop M, Waddington J, Harden J, et al.. (2008) Short-term response of methane fluxes and methanogen activity to water table and soil warming manipulations in an Alaskan peatland. Journal of Geophysical Research: Biogeosciences (2005–2012) 113.

[66] Chivers M, Turetsky M, Waddington J, Harden J, McGuire A (2009) Effects of experimental water table and temperature manipulations on ecosystem CO2 fluxes in an Alaskan rich fen. Ecosystems 12: 1329–1342.

[67] Guo Y, Wan Z, Liu D (2010) Dynamics of dissolved organic carbon in the mires in the Sanjiang Plain, Northeast China. Journal of Environmental Sciences 22: 84–90. 10.1016/s1001-0742(09)60078-4 20397391

[68] Walling DE, Webb BW (1981) The reliability of suspended sediment load data: IAHS Publication.

[69] Clair TA, Arp P, Moore TR, Dalva M, Meng FR (2002) Gaseous carbon dioxide and methane, as well as dissolved organic carbon losses from a small temperate wetland under a changing climate. Environmental Pollution 116 Supplement 1S143–S148. 11833902 10.1016/s0269-7491(01)00267-6

[70] Weltzin JF, Pastor J, Harth C, Bridgham SD, Updegraff K, et al (2000) Response of bog and fen plant communities to warming and water-table manipulations. Ecology 81: 3464–3478.

[71] Fraser C, Roulet N, Moore T (2001) Hydrology and dissolved organic carbon biogeochemistry in an ombrotrophic bog. Hydrological Processes 15: 3151–3166.

[72] Moore T (1989) Dynamics of dissolved organic carbon in forested and disturbed catchments, Westland, New Zealand: 1. Maimai. Water Resources Research 25: 1321–1330.

[73] Urban N, Bayley S, Eisenreich S (1989) Export of dissolved organic carbon and acidity from peatlands. Water Resources Research 25: 1619–1628.

[74] Worrall F, Reed M, Warburton J, Burt T (2003) Carbon budget for a British upland peat catchment. Science of The Total Environment 312: 133–146. 12873406 10.1016/S0048-9697(03)00226-2

[75] Steinberg (2003) Ecology of humic substances in freshwaters: determinants from geochemistry to ecological niches. Berlin: Springer.

[76] Clymo RS (1984) The limits to peat bog growth. Philosophical Transactions of the Royal Society of London B, Biological Sciences 303: 605–654.

[77] Mars H, Wassen MJ, Peeters WHM (1996) The effect of drainage and management on peat chemistry and nutrient deficiency in the former Jegrznia-floodplain (NE-Poland). Vegetatio 126: 59–72.

[78] Freeman C, Ostle N, Kang H (2001) An enzymic 'latch' on a global carbon store. Nature 409: 149–149. 10.1038/35051650 11196627

[79] Moore PD (2002) The future of cool temperate bogs. Environmental Conservation 29: 3–20.

[80] Strakova P, Penttilä T, Laine J, Laiho R (2012) Disentangling direct and indirect effects of water table drawdown on above-and belowground plant litter decomposition: consequences for accumulation of organic matter in boreal peatlands. Global Change Biology 18: 322–335.

[81] Whittington PN, Price JS (2006) The effects of water table draw-down (as a surrogate for climate change) on the hydrology of a fen peatland, Canada. Hydrological Processes 20: 3589–3600.

[82] Harrison AF, Taylor K, Scott A, Poskitt J, Benham D, et al (2008) Potential effects of climate change on DOC release from three different soil types on the Northern Pennines UK: examination using field manipulation experiments. Global Change Biology 14: 687–702.

[83] Froberg M, Berggren D, Bergkvist B, Bryant C, Mulder J (2006) Concentration and fluxes of dissolved organic carbon (DOC) in three norway spruce stands along a climatic gradient in sweden. Biogeochemistry 77: 1–23.

[84] Lin X, Zhang Z, Wang S, Hu Y, Xu G, et al (2011) Response of ecosystem respiration to warming and grazing during the growing seasons in the alpine meadow on the Tibetan plateau. Agricultural and Forest Meteorology 151: 792–802.

[85] Kim SY, Freeman C, Fenner N, Kang H (2012) Functional and structural responses of bacterial and methanogen communities to 3-year warming incubation in different depths of peat mire. Applied Soil Ecology 57: 23–30.

