# Supplementary figures and images for: Rapid Response of Hydrological Loss of DOC to Water Table Drawdown and Warming in Zoige Peatland: Results from a Mesocosm Experiment
Source: PLoS One. 2014 Nov 4;9(11):e109861. doi: 10.1371/journal.pone.0109861 (PMC4219674; doi:10.1371/journal.pone.0109861)

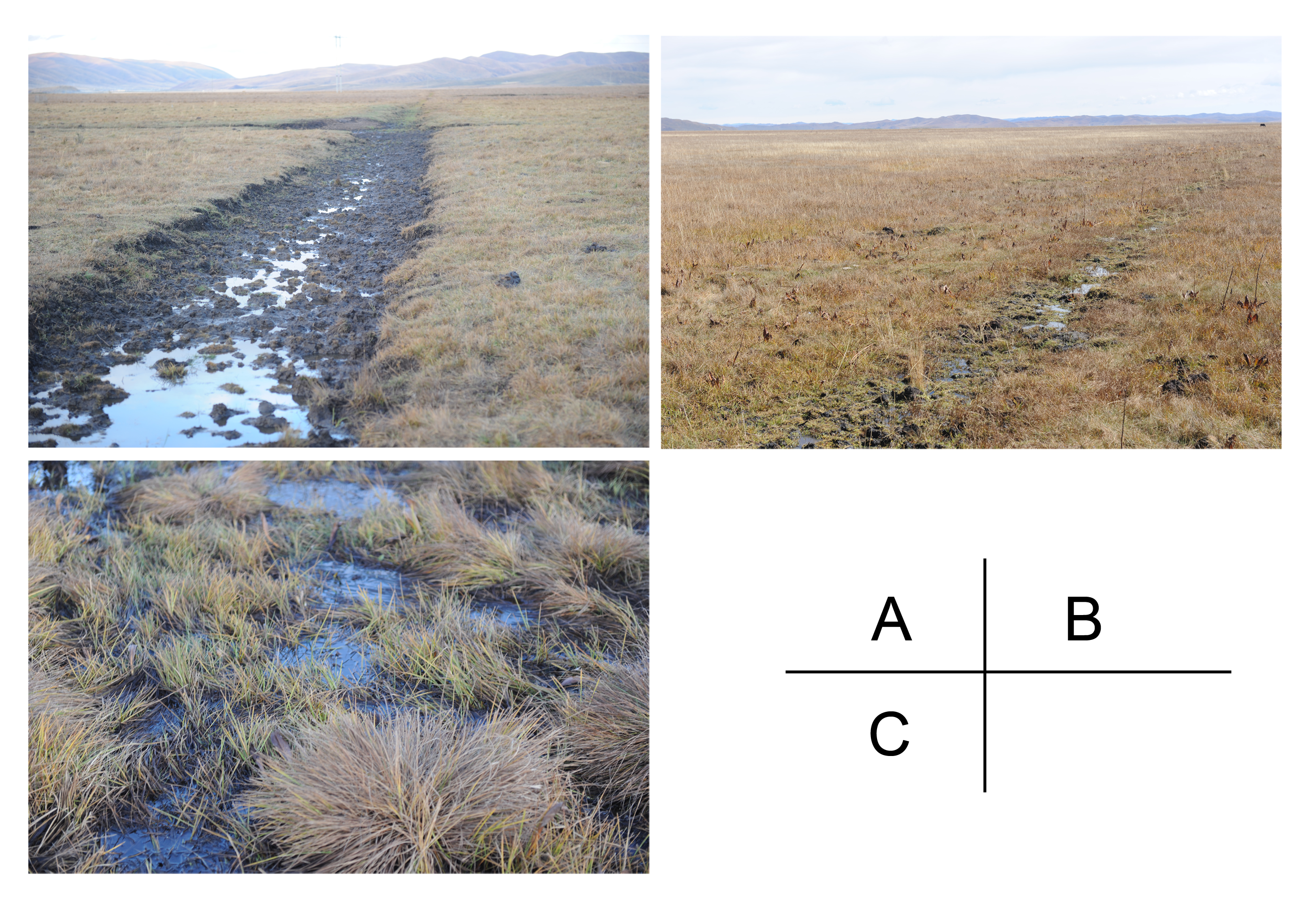

Supplement: Figure S1 — Topography and vegetation characteristics of the study area. (A) Drainage ditch. (B) Vegetation community growing in shallow water. (C) Scattered vegetation surrounded by surface water. All photographs were taken during May 2012 in Hongyuan County located in Zoige peatland. (TIF) [file pone.0109861.s001.tif]

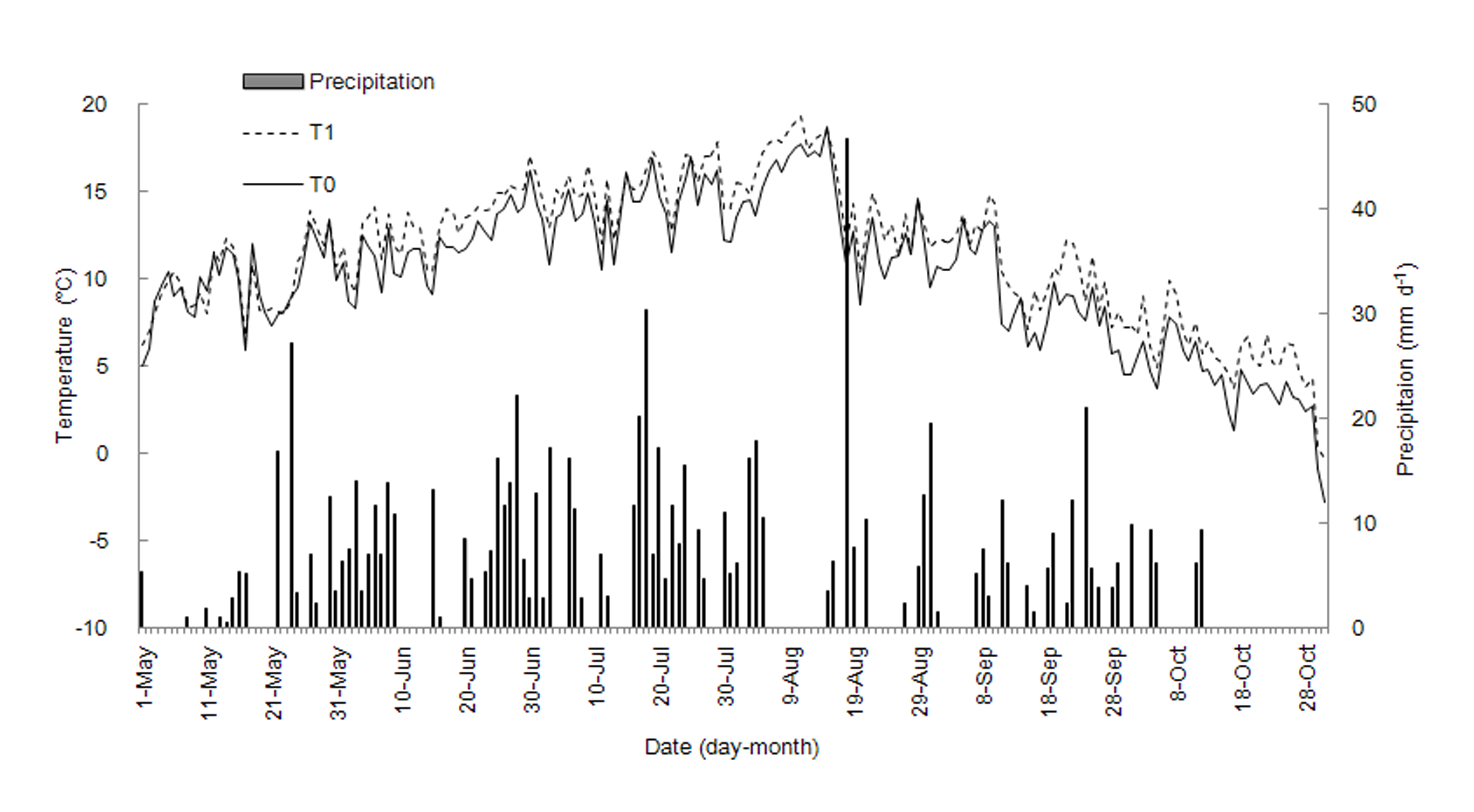

Supplement: Figure S2 — Peat temperature at −10 cm depth and precipitation during the growing season in 2012. (TIF) [file pone.0109861.s002.tif]

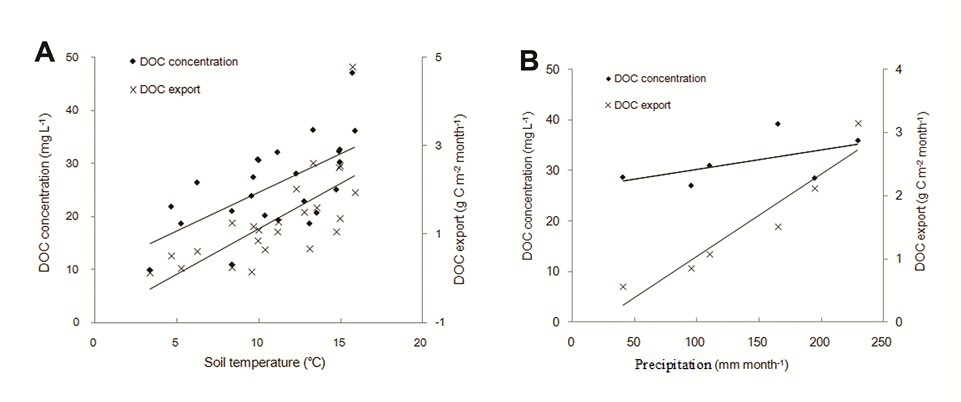

Supplement: Figure S3 — Correlation analysis of temperature and precipitation with DOC. (A) Results of the correlation analysis between peat temperature recorded in four mesocosms and corresponding mean monthly export and concentration of DOC (DOC concentration: y = 1.4573x +9.9416, R 2 = 0.4025, p<0.01, n = 24; DOC export: y = 0.2043x −0.9177, R 2 = 0.4984, p<0.01, n = 24). (B) Results of the correlation analysis between mean monthly precipitation and mean monthly export and concentration of DOC in all mesocosms (DOC concentration: y = 0.0379x +26.409, R 2 = 0.3046, p = 0.128, n = 6; DOC export: y = 0.013x −0.2987, R 2 = 0.8982, p<0.01, n = 6). (TIF) [file pone.0109861.s003.tif]

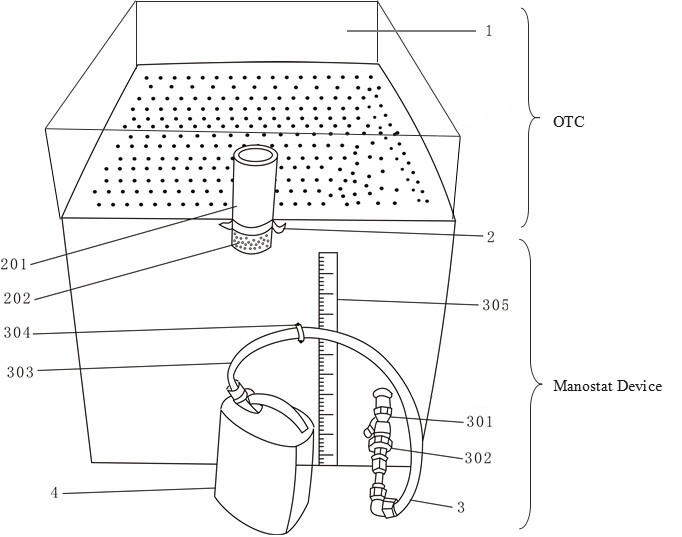

Supplement: Figure S4 — The schematic drawing of the mesocosm in the study. The references of the number are shown as below: 1. Polycarbonate solar panels; 2. Water intake system; 3. Drainage system; 4. Water storage barrel; 201. Observation tube of water-level; 202. Sand filter pocket of inlet; 301. Sand filter pocket of outlet; 302. High pressure valves; 303. Water pipe; 304. Hanger loop; 305. Observation rule. (TIF) [file pone.0109861.s004.tif]
